# Health-related quality of life in patients with newly diagnosed inflammatory bowel disease: an observational prospective cohort study (IBSEN III)

**DOI:** 10.1007/s11136-023-03435-9

**Published:** 2023-05-23

**Authors:** Bjorn Christian Olsen, Randi Opheim, Vendel A. Kristensen, Marte Lie Høivik, Charlotte Lund, Tone Bergene Aabrekk, Ingunn Johansen, Kristina Holten, Vibeke Strande, May-Bente Bengtson, Petr Ricanek, Trond Espen Detlie, Tomm Bernklev, Lars-Petter Jelsness-Jørgensen, Gert Huppertz-Hauss

**Affiliations:** 1 Department of Gastroenterology, Skien Hospital, Telemark Hospital Trust, Ulefossvegen 55, 3710 Skien, Norway; 2 Institute of Clinical Medicine, University of Oslo, Oslo, Norway; 3 Department of Gastroenterology, Oslo University Hospital, Oslo, Norway; 4 Department of Nursing Science, Institute of Health and Society, University of Oslo, Oslo, Norway; 5 Unger-Vetlesen Institute, Lovisenberg Diaconal Hospital, Oslo, Norway; 6 Vestfold Hospital Trust, Research and Development, Tønsberg, Norway; 7 Faculty of Health, Welfare and Organisation, Østfold University College, Halden, Norway; 8 Department of Gastroenterology, Østfold Hospital Trust, Sarpsborg, Norway; 9 Department of Gastroenterology, Akershus University Hospital, Lørenskog, Norway

**Keywords:** Health-related quality of life, Inflammatory bowel disease, Short Form 36, Norwegian Inflammatory Bowel Disease Questionnaire

## Abstract

**Purpose:**

This unselected, population-based cohort study aimed to determine the level of health-related quality of life (HRQoL) in patients with Crohn’s disease (CD) and ulcerative colitis (UC) at the time of diagnosis compared with a reference population and identify the demographic factors, psychosocial measures, and disease activity markers associated with HRQoL.

**Methods:**

Adult patients newly diagnosed with CD or UC were prospectively enrolled. HRQoL was measured using the Short Form 36 (SF-36) and Norwegian Inflammatory Bowel Disease Questionnaires. Clinical significance was assessed using Cohen’s *d* effect size and further compared with a Norwegian reference population. Associations between HRQoL and symptom scores, demographic factors, psychosocial measures, and disease activity markers were analyzed.

**Results:**

Compared with the Norwegian reference population, patients with CD and UC reported significantly lower scores in all SF-36 dimensions, except for physical functioning. Cohen’s *d* effect sizes for men and women in all SF-36 dimensions were at least moderate, except for bodily pain and emotional role for men with UC and physical functioning for both sexes and diagnoses. In the multivariate regression analysis, depression subscale scores ≥ 8 on the Hospital Anxiety and Depression Scale, substantial fatigue, and high symptom scores were associated with reduced HRQoL.

**Conclusion:**

Patients newly diagnosed with CD and UC reported statistically and clinically significantly lower scores in seven of the eight SF-36 dimensions than the reference population. Symptoms of depression, fatigue, and elevated symptom scores were associated with poorer HRQoL.

## Plain English summary

Previous research has shown that patients with inflammatory bowel disease often experience reduced health-related quality of life later in the course of the disease, although little is known about their quality of life at the time of diagnosis. In our study, we compared the health-related quality of life of an unselected group of newly diagnosed patients with inflammatory bowel disease with that of a representative sample of the Norwegian reference population. We also evaluated various social, psychological, and disease-related factors that may be associated with reduced health-related quality of life. Our results showed that newly diagnosed patients with inflammatory bowel disease experienced low health-related quality of life compared with the Norwegian reference population, and that depression, fatigue, and bowel symptoms (e.g., diarrhea) contributed the most among the factors evaluated. Surprisingly, and contrary to our expectations, high levels of fecal calprotectin, a protein that indicates the grade of bowel inflammation, weakly corresponded to reduced health-related quality of life. Ongoing research on the same patient group may likely reveal whether these patterns change over time. The results of this study can help healthcare providers identify patients with low health-related quality of life early in the course of their inflammatory bowel disease, which may lead to more timely and effective interventions.

## Introduction

Crohn’s disease (CD) and ulcerative colitis (UC) are chronic inflammatory bowel diseases (IBD) that are associated with reduced health-related quality of life (HRQoL) [1, 2]. Although clinical remission and endoscopic mucosal healing remain the primary goals of treatment for CD and UC, improvement of HRQoL has become an increasingly important goal and recently included in the Selecting Therapeutic Targets in Inflammatory Bowel Disease II treatment target recommendations [3]. Many cross-sectional and longitudinal studies have shown that the HRQoL of patients with IBD improves over time with treatment [4]. However, most published studies to date are based on selected patient cohorts or pharmaceutical trials and do not include newly diagnosed patients. Therefore, data on HRQoL in newly diagnosed, unselected patients are limited [4, 5]. To our knowledge, only two previous studies have reported baseline data on HRQoL in an unselected population-based IBD cohort [5, 6]. The lack of HRQoL data at the time of diagnosis in an unselected IBD population cohort makes the evaluation of future HRQoL measurements difficult and limits the accurate interpretation of treatment effects on HRQoL over time.

This study primarily aimed to compare HRQoL in patients newly diagnosed with IBD with that in a representative sample of the Norwegian population. We hypothesized that the HRQoL of newly diagnosed patients would be significantly lower than that of the reference population. The secondary aim was to identify demographic factors, psychosocial measures, and objective disease activity markers associated with HRQoL scores prior to the initiation of IBD treatment.

## Materials and methods

### Study population

The Inflammatory Bowel Disease in Southeastern Norway III (IBSEN III) is a population-based IBD inception cohort study that included newly diagnosed adult and pediatric patients from the South-Eastern Health Region of Norway (Clinical Trials ID: NCT02727959). Its overall design and study scope are described in detail elsewhere [7]. In the IBSEN III study, patients provided demographic information, social data, and blood and stool samples for disease marker analysis. Additionally, they were asked to complete patient-reported outcome measures (PROMs), including HRQoL questionnaires. All patients underwent physical examination and diagnostic ileocolonoscopy with biopsy. Magnetic resonance imaging (MRI) of the small bowel was performed in patients with suspected CD. For the purposes of this study, we defined the study population as adult patients (aged ≥ 18 years) from the three largest hospitals in the IBSEN III study: Oslo University Hospital, Akershus University Hospital, and Vestfold Hospital Trust. All patients provided written informed consent before inclusion in both the IBSEN III and current studies. The diagnostic criteria for adults with CD and UC used in this study were based on the internationally accepted Lennard–Jones Criteria [8] and adapted from Moum et al. [9].

### Data collection

Patients diagnosed with CD or UC from January 1, 2017 to December 31, 2019 were included by the researchers local to each of the three participating hospitals. Clinical and demographic data were recorded in the electronic case report form (eCRF) system Viedoc© (PCG Solutions AB, St Persgatan 6, 753 20 Uppsala, Sweden), which is approved for the collection and storage of research data in Norway. Patients received access to an internet-based system (ViedocMe©) and were asked to complete PROMs.

Central study investigators checked the eCRF data for completeness and accuracy and compared the results with local hospital patient records to supplement eCRF data and adjust for obvious imputation errors. Missing eCRF data were only included if the central study investigators located the relevant data in the local hospital patient records and could further attest its validity for the study purposes. Ambiguous or common irregularities were reviewed by a panel of IBSEN III investigators and adjusted if a consensus was reached. Otherwise, the eCRF data remained unaltered.

### Demographic and social data

Marital status was dichotomized into living together (married/co-inhabitant) or alone (single, widowed, separated/divorced). Educational status was based on the Norwegian educational system and dichotomized into higher (> 12 years attended and at least two years of university) or basic (≤ 12 years) education only. Employment status was dichotomized into employed/studying or non-employed/not studying (homemaker, disability beneficiary, unemployed, or retired). Current smoking status was categorized as yes for patients who smoked one year prior to diagnosis or later and no for those who did not.

### Disease activity

Generally accepted clinical measures of disease activity are the Harvey–Bradshaw Index (HBI) for CD [10] and Simple Clinical Colitis Activity Index (SCCAI) for UC [11]. A score of ≥ 5 on the HBI and ≥ 2.5 on the SCCAI was defined as active disease for CD and UC, respectively [12, 13]. Blood samples were collected for the analysis of C-reactive protein (CRP), and a cutoff value of ≥ 5 was considered elevated. Fecal stool samples were collected for calprotectin analysis using an enzyme-linked immunoassay (Bühlmann Calprotectin ELISA EK-CAL; Bühlmann Laboratories AG, Switzerland). Samples < 30 µg/g were registered as 29 µg/g and > 1800 µg/g as 1801 µg/g. A value > 250 µg/g represented active IBD inflammation [14, 15].

For UC, the extent of colonic disease was categorized using Montreal endoscopic subscores [16], and the severity of inflammation was classified using Mayo endoscopic subscores [17]. Furthermore, the Montreal criteria were applied to categorize overall UC disease severity into remission, mild UC, moderate UC, or severe UC [16].

For CD, complicated CD was defined as cases with penetrating disease, perianal disease, and/or strictures, similar to the definition used by Burisch et al. [18].

### HRQoL questionnaires

#### The Norwegian Inflammatory Bowel Disease Questionnaire (N-IBDQ)

The N-IBDQ is a disease-specific HRQoL questionnaire consisting of 32 questions divided into five dimensions: emotional function (E1), stool consistency and pattern (B1), bowel pain and discomfort (B2), social function (S1), and worry (E2). The total score was calculated, yielding a score of 32–224 points, with higher scores indicating better HRQoL [19]. The N-IBDQ was translated into Norwegian and validated in an earlier IBSEN cohort [20]. Missing data were added, as previously described by Bernklev et al. [20]. If half or more of the questions in a particular dimension were answered, the missing values were replaced by the respondent’s mean score for the remaining answers in that dimension [20]. If less than half of the questions were answered, the dimension was left as it was, and the total N-IBDQ score was not calculated.

#### The Short Form 36 (SF-36)

The SF-36 is a generic HRQoL questionnaire consisting of 36 questions divided into eight dimensions: physical functioning (PF), role limitation due to physical health (RP), bodily pain (BP), general health (GH), vitality (VT), social functioning (SF), role limitations due to emotional health (RE), and mental health (MH). Each dimension yields a score of 0–100, with higher scores indicating better HRQoL [21]. The missing data were supplemented as recommended by the original SF-36 manual by Ware et al. [21]. If half or more of the questions in a particular dimension were answered, the missing values were replaced with the respondent’s mean score for the remaining answers in that dimension [21]. If less than half of the questions were answered, the dimension was left as it was.

The SF-36 has been translated into Norwegian and previously validated in the Norwegian population [22]. The reference population used in our study was obtained from a normative study consisting of 2,323 Norwegian citizens aged 19–80 years who were randomly selected from the Norwegian National Population Register [22]. Among these, 51% were female and 49% were male. The mean age of the sample was 44.9 years with a standard deviation (SD) of 16.5. Both crude, sex-stratified SF-36 scores and scores adjusted for education level, sex, and age were utilized, as previously recommended [23]. We chose this reference population due to its acceptable response rate of 67% and a more similar mean age (44.9 years) to our study cohort (38.5 years, SD 14.5) than other SF-36 studies of the Norwegian population [24]. Furthermore, the SF-36 scores in the reference population have been shown to be stable over time [24].

#### Additional questionnaires

In addition to HRQoL, patients were asked to complete the Hospital Anxiety and Depression Scale (HADS), General Self-Efficacy Scale (GSE), and Fatigue Questionnaire (FQ). For the HADS, subscale scores for anxiety (HADS-A) and depressive symptoms (HADS-D) ≥ 8 were considered possible cases of anxiety or depression, respectively [25]. For GSE, the total scores in this study were analyzed [26]. For FQ, a total dichotomized score ≥ 4 indicated substantial fatigue [27]. Further, a total FQ score ≥ 4 in combination with a duration of six months or more indicated chronic fatigue. All the above-mentioned questionnaires were translated into Norwegian and previously validated in the Norwegian population [28–30].

### Statistics

Demographic factors and disease characteristics at baseline are presented as medians and interquartile ranges, mean values with SD, or percentages, when appropriate. Data from patients with CD and UC were stratified by sex, and the results are presented separately. Continuous data were assessed using parametric methods (independent samples t-test) when normally distributed and non-parametric methods (Mann–Whitney U test) when skewed. Categorical data were analyzed using the Chi-square test.

The SF-36 and N-IBDQ dimensional scores were checked for normality and adjusted for age, sex, and educational status using analysis of covariance. Mean scores were stratified by sex and presented as SD or 95% confidence intervals (CI), when appropriate. Dimensional SF-36 scores from the reference population are presented as adjusted mean scores with SD [22]. Differences in SF-36 scores between the study and reference populations were determined by calculating Cohen’s *d* effect sizes ([mean patient scor e− mean reference population score]/pooled SD), where < 0.2 indicated no difference, 0.2–0.5 indicated a small difference, 0.5–0.8 indicated a moderate difference and > 0.8 indicated a large difference [31]. A moderate-to-large difference was considered clinically important [32]. Homogeneity of variance in the study cohort and reference population were assumed. The N-IBDQ scores are presented as both dimensional and total scores.

Multiple linear regression (blockwise enter method) was performed to evaluate the demographic factors, psychosocial measures, disease activity markers, and clinical variables associated with the SF-36 dimensional and N-IBDQ total scores. Variables for the multiple regression model were selected after performing a univariate analysis of variables known to influence HRQoL and chosen by the investigators. All regression analyses were performed separately for patients with CD and UC. Age, gender, completed education (basic education only), employment status (not employed/not studying), disease biomarkers (calprotectin > 250 µg/g, CRP ≥ 5 mg/L) and variables with *p* values < 0.2 in more than two dimensions from the univariate analysis were included in the multiple regression model. Unstandardized regression coefficients (β) are reported with 95% CI. The results from the multiple regression were checked for collinearity and model fit, and the residuals were analyzed. Due to multiple testing, the significance level was set to *p* < 0.01 in all analyses of HRQoL and multivariate regression. Variables with insignificant β coefficients for all HRQoL dimensions in multiple regression remained in the model, but are not presented. All statistical analyses were performed using IBM SPSS Statistics for Windows, version 28 (IBM Corp., Armonk, N.Y., USA).

## Ethics

This study was approved by the Southeast Regional Committee for Medical Research Ethics (REC South East) (ref. 2015/946-3) and Norwegian Center for Research Data (NSD ref. 498873). Study inclusion did not imply any changes in patient treatment, and all patients provided written informed consent. The study was conducted in accordance with the principles of the Declaration of Helsinki.

## Results

**Fig. 1:**
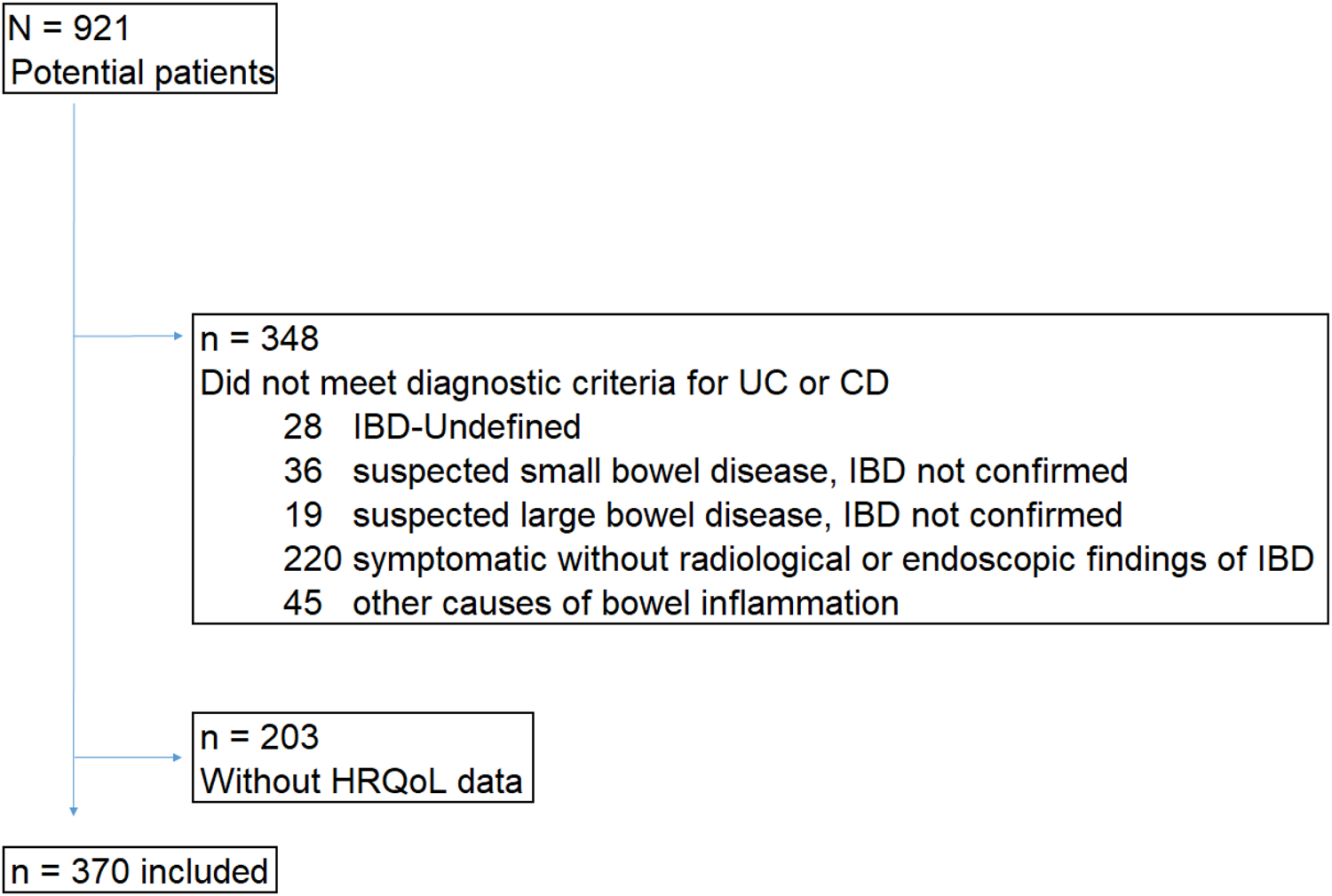
Flowchart showing the included and excluded participants in the study. *UC* ulcerative colitis, *CD* Crohn’s disease, *IBD* inflammatory bowel disease, *HRQoL* health-related quality of life

A total of 921 patients were recruited from the three centers from January 1, 2017 to December 31, 2019. Among them, 198 (21.5%) and 375 (40.7%) met the predefined diagnostic criteria for CD and UC, respectively. PROM data on HRQoL were available for 370 patients (64.5%) who constituted the study population (CD, 131 (35.4%); UC, 239 (64.6%)) (Fig. 1).

Among the 370 patients who provided HRQoL data, missing SF-36 data were supplemented in 25 patients (6.8%), with an average of one missing answer per patient (range, 0–4), which was considered low. Significant patterns in missing SF-36 data were not found. For the N-IBDQ, missing data were supplemented in 47 patients (12.7%), with 39 patients (10.5%) missing only one or two answers. To determine whether the study population was representative, the demographics, psychological factors, and clinical disease markers were compared between patients who answered the N-IBDQ questionnaire and those who did not. Except for patients with UC with Mayo endoscopic score ≥ 2 who answered the N-IBDQ questionnaire more frequently (83.9% vs. 72.4%, *p* = 0.01), no differences were observed between the two groups. Notably, the median Mayo endoscopic scores in both groups were equal, suggesting that this difference was not clinically important.

The demographics, psychosocial measures, and disease characteristics of the study population at baseline are presented in Table 1. No statistically significant differences were observed between patients with CD and UC in terms of age, marital status, educational status, or employment status. Compared with patients with UC, more patients with CD were women (58.0% vs 46.4%, *p* = 0.039), smokers (23.5% vs 14.4%, *p* = 0.021), had substantial fatigue (72.8% vs 61.4%, *p* = 0.036), experienced longer symptom duration before diagnosis (median 12 months vs 4 months, *p* < 0.001), had a higher median HADS-D score (4 vs 3, *p* = 0.034), and a higher median CRP (4.4 mg/L vs 1.9 mg/L, *p* < 0.001). Complicated CD was found in 23.7% of patients with CD. Most patients with UC presented with proctitis (40.6%) and moderate Montreal disease severity (52.3%). Median calprotectin levels were similar in both patient groups (CD: 369 µg/g vs UC: 242 µg/g, *p* = 0.307).

**Table 1:** Demographics, psycho-social measures, and disease characteristics at the time of diagnosis

	Ulcerative colitis (UC)	Crohn’s disease (CD)	*p* value*
	n = 239 (64.6%)	n = 131 (35.4%)	
*Demographics*			
Age, mean years (SD)	38 (14)	39 (15)	0.428
Gender			0.039
Female (% of n)	111 (46.4%)	76 (58.0%)	
Male (% of n)	128 (53.6%)	55 (42.0%)	
Marital status			0.171
Single	67 (28.0%)	41 (31.3%)	
Married/cohabitant	163 (68.2%)	81 (61.8%)	
Separated/divorced	9 (3.8%)	7 (5.3%)	
Widowed	0 (0.0%)	2 (1.5%)	
Completed education			0.708
Primary school, ≤ 10 years (% of n)	21 (8.8%)	12 (9.2%)	
Secondary school, > 10 ≤ 12 years (% of n)	92 (38.5%)	56 (42.7%)	
University, at least two years (% of n)	126 (52.7%)	63 (48.1%)	
Employment status			0.060
Employed (% of n)	180 (75.3%)	80 (61.5%)	
Student	32 (13.4%)	28 (21.5%)	
Homemaker	2 (0.8%)	0 (0.0%)	
Disability beneficiary	10 (4.2%)	10 (7.7%)	
Unemployed	7 (2.9%)	4 (3.1%)	
Retired	8 (3.3%)	8 (3.3%)	
Current smoking (%)^a^	16 (14.4%)	16 (23.5%)	0.021
*Psycho-social measures*			
HADS-Anxiety, median value (range)	5 (0–19)	6 (0–18)	0.125
HADS-Depression, median value (range)	3 (0–18)	4 (0–18)	0.034
General self-efficacy, median value (range)	31 (12–40)	30 (12–40)	0.397
Substantial fatigue^b^	143 (61.4%)	91 (72.8%)	0.036
*Disease characteristics*			
Symptom duration, median months (range)	4 (1–120)	12 (0–240)	< 0.001
HBI for CD, median value (range)	–	4 (0–25)	
Complicated CD (% of n)	–	31 (23.7%)	
SCCAI for UC, median value (range)	4 (0–12)	–	
Mayo endoscopic subscore for UC (% of n)			
Normal or inactive	0 (0.0%)	–	
Mild	38 (16.1%)	–	
Moderate	149 (63.1%)	–	
Severe	49 (20.8%)	–	
Montreal endoscopic subscore for UC (% of n)			
Ulcerative proctitis (E1)	97 (40.6%)	–	
Left-sided UC (E2)	52 (21.8%)	–	
Extensive UC (E3)	90 (37.7%)	–	
Montreal disease severity for UC (% of n)			
Clinical remission (S0)	4 (1.7%)	–	
Mild UC (S1)	89 (37.2%)	–	
Moderate UC (S2)	125 (52.3%)	–	
Severe UC (S3)	21 (8.8%)	–	
Calprotectin, median value (range)	242 (29–1801)	369 (29–1801)	0.307
CRP, median value (range)	1.9 (0.6–119)	4.4 (0.6–270)	< 0.001

*SD* standard deviation, *HADS* Hospital Anxiety and Depression Scale, *HBI* Harvey–Bradshaw Index, *SCCAI* Simple Clinical Colitis Activity Index

*Chi-square tests were performed for categorical variables, independent samples t-tests for normally distributed continuous variables, and Mann–Whitney U-tests for continuous variables not normally distributed. A *p* value < 0.05 represented a statistically significant difference between patients with UC and CD

^a^Of patients who report having previously smoked (111 patients with UC, 68 patients with CD)

^b^Defined as a total score ≥ 4 on the Fatigue Questionnaire

### Generic HRQoL

The SF-36 dimensional mean scores stratified by sex and diagnosis are presented in Table 2. Women with UC had significantly lower mean scores on the BP, VT, and MH dimensions than men with UC. Women with CD reported significantly lower VT scores than men (Table 2).

**Table 2:** SF-36 and N-IBDQ dimensional scores, stratified by diagnosis and gender, and compared with a Norwegian reference population

	Ulcerative colitis (UC)			Crohn’s disease (CD)			Norwegian reference population (N = 2323)		
	Male (n = 128)^b^	Female (n = 111)^b^	All UC (n = 239)^a^	Male (n = 55)^b^	Female (n = 76)^b^	All CD (n = 131)^a^	Male (n = 1072–1127)^c^	Female (n = 1110–1184)^c^	All^a^
*SF-36*									
Physical function (PF)	87.0 (83.9–90.2)	84.6 (81.2–88.0)	85.8 (83.5–88.1)	86.9 (82.1–91.8)	82.9 (78.8–87.0)	84.8 (81.7–88.0)	89.8 (15.5)	84.8 (20.8)	88.3 (17.4)
Role physical (RP)	56.1 (48.7–63.6)^*^	44.5 (36.4–52.5)^#^	50.2 (44.8–55.7)	57.0 (45.9–68.1)^*^	45.1 (35.8–54.5)^#^	50.0 (42.6–57.3)	80.5 (33.6)	75.4 (37.7)	79.5 (34.4)
Bodily pain (BP)	67.6 (63.0–72.2)^†*^	56.6 (51.7–61.5)^†#^	62.1 (58.7–65.4)	57.7 (50.7–64.7)^*^	50.2 (44.3–56.2)^#^	54.1 (49.6–58.6)	77.2 (25.0)	73.0 (26.6)	75.6 (25.6)
General health (GH)	64.5 (60.5–68.5)^*^	57.7 (53.3–62.0)^#^	61.0 (58.1–64.0)	58.6 (52.5–64.8)^*^	48.8 (43.5–54.0)^#^	53.5 (49.5–57.5)	77.4 (21.3)	76.3 (22.5)	77.2 (21.8)
Vitality (VT)	44.4 (40.5–48.3)^†*^	34.6 (30.4–38.7)^†#^	39.4 (36.6–42.2)	43.3 (37.4–49.2)^†*^	33.1 (28.1–38.1)^†#^	38.1 (34.3–41.9)	63.2 (19.9)	56.9 (21.2)	60.3 (20.7)
Social functioning (SF)	72.0 (67.0–76.9)^*^	63.2 (57.8–68.5)^#^	67.5 (63.9–71.1)	67.7 (60.2–75.2)^*^	59.2 (52.8–65.7)^#^	63.4 (58.5–68.3)	87.6 (20.9)	83.7 (23.1)	86.1 (21.8)
Role emotional (RE)	68.0 (60.7–75.4)^*^	57.1 (49.2–65.0)^#^	62.5 (57.1–67.9)	65.8 (54.6–77.0)^*^	56.0 (46.5–65.6)^#^	60.9 (53.6–68.2)	84.5 (29.7)	79.1 (34.6)	82.5 (31.8)
Mental health (MH)	71.3 (68.1–74.5)^†*^	62.4 (59.0–65.9)^†#^	66.8 (64.5–69.2)	69.7 (64.8–74.6)^*^	62.4 (58.3–66.6)^#^	66.1 (62.9–69.3)	80.0 (15.8)	77.6 (17.0)	78.7 (16.4)
*N-IBDQ*									
Emotional function (E1)	54.2 (51.9–56.4)^†^	49.3 (46.9–51.6)^†^	51.7 (50.0–53.3)	52.8 (49.4–56.1)	46.8 (43.9–49.6)	49.7 (47.5–51.9)			
Stool consistency and pattern (B1)	36.3 (34.9–37.7)	36.3 (34.8–37.7)	36.3 (35.3–37.3)	40.4 (38.3–42.5)	38.5 (36.7–40.3)	39.4 (38.0–40.7)			
Bowel pain and discomfort (B2)	25.0 (23.8–26.2)^†^	22.5 (21.2–23.8)^†^	23.7 (22.8–24.6)	23.5 (21.6–25.4)	20.8 (19.2–22.4)	22.2 (20.9–23.4)			
Social function (S1)	21.3 (20.2–22.4)	20.6 (19.4–21.8)	20.9 (20.1–21.7)	21.5 (19.8–23.2)	20.8 (19.4–22.3)	21.2 (20.1–22.3)			
Worries (E2)	26.4 (25.4–27.5)	25.8 (24.6–26.9)	26.1 (25.3–26.9)	26.2 (24.6–27.8)	25.2 (23.8–26.5)	25.7 (24.6–26.7)			
N-IBDQ total score	163.1 (157.4–168.8)	154.4 (148.2–160.5)	158.7 (154.5–162.8)	164.4 (155.7–173.1)	152.1 (144.7–159.5)	158.0 (152.4–163.7)			

SF-36, Short Form 36; N-IBDQ, Norwegian Inflammatory Bowel Disease Questionnaire

^a^Mean scores adjusted for age, gender and educational status with 95% confidence intervals in parentheses are shown

^b^Mean scores adjusted for age and educational status with 95% confidence intervals in parentheses are shown

^c^crude, unadjusted mean scores with standard deviations (SD). Since the total number of respondents for each SF-36 dimension varied, the range of responses is reported

^†^Significant difference between men and women with same diagnosis, *p* < 0.01

*Significant difference between patients and reference population in males, *p* < 0.01

^#^Significant difference between patients and reference population in females, *p* < 0.01

**Fig. 2:**
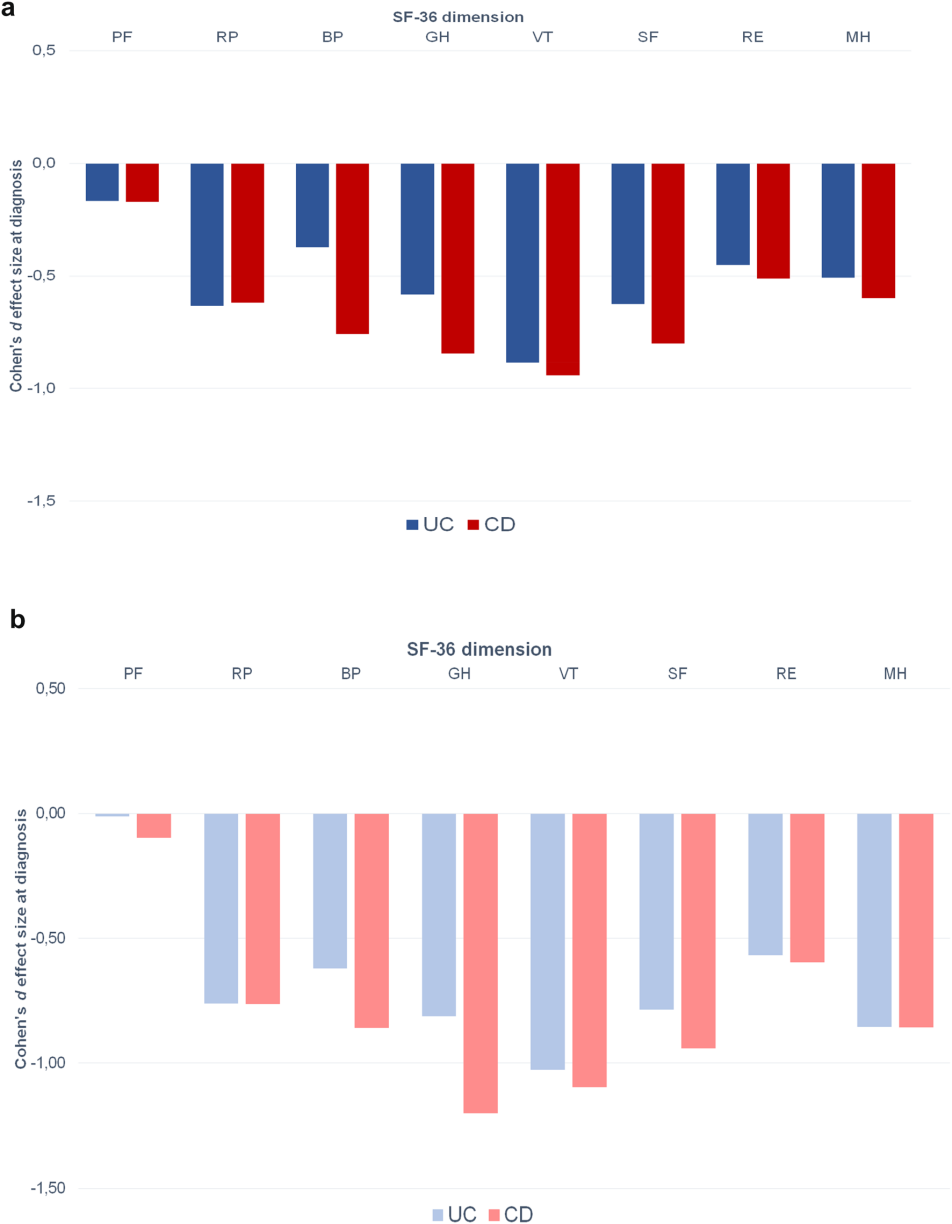
**a** Cohen’s *d* effect size in each Short Form 36 dimension for study men compared with men in the Norwegian reference population, stratified by diagnosis. **b** Cohen’s *d* effect size in each Short Form 36 dimension for study women compared with women in the. Norwegian reference population, stratified by diagnosis.** a**, **b** In accordance with the Cohen’s effect size index where < 0.2 indicated no difference, 0.2–0.5 indicated a small difference, 0.5–0.8 indicated a moderate difference, and > 0.8 indicated a large difference (Cohen J. *Statistical power analysis for the behavioral sciences*. 2nd ed. Hillsdale, NJ: Laurence Erlbaum Associates; 1988)

Both men and women with CD and UC reported significantly lower scores than the reference population in all SF-36 dimensions, except in PF (Table 2). Figure 2a, show the Cohen’s *d* effect sizes for the study patients compared with the reference population. Apart from BP and RE for men with UC and PF for both sexes and diagnoses, a moderate effect size (> 0.5) was found for men and women in all SF-36 dimensions. For women with CD, a large effect size (> 0.8) was observed in the BP, GH, VT, SF, and MH dimensions, whereas a large effect size was observed in the GH, VT, and SF dimensions in men with CD (Fig. 2a, b). For women with UC, large effect sizes were found in GH, VT, and MH, whereas only VT displayed a large effect size in men with UC (Fig. 2a, b).

**Fig. 3:**
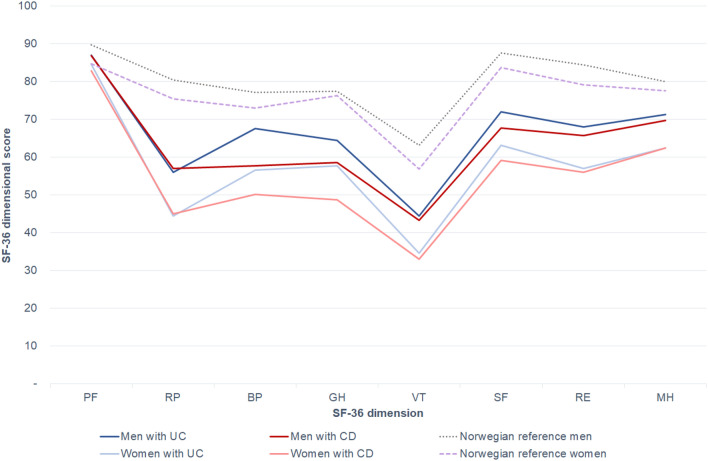
Mean Short Form 36 dimensional scores in study patients compared with a Norwegian reference population, stratified by gender and diagnosis

SF-36 dimensional mean scores for the study and reference population stratified by sex are presented in Fig. 3.

### Disease-specific HRQoL

**Fig. 4:**
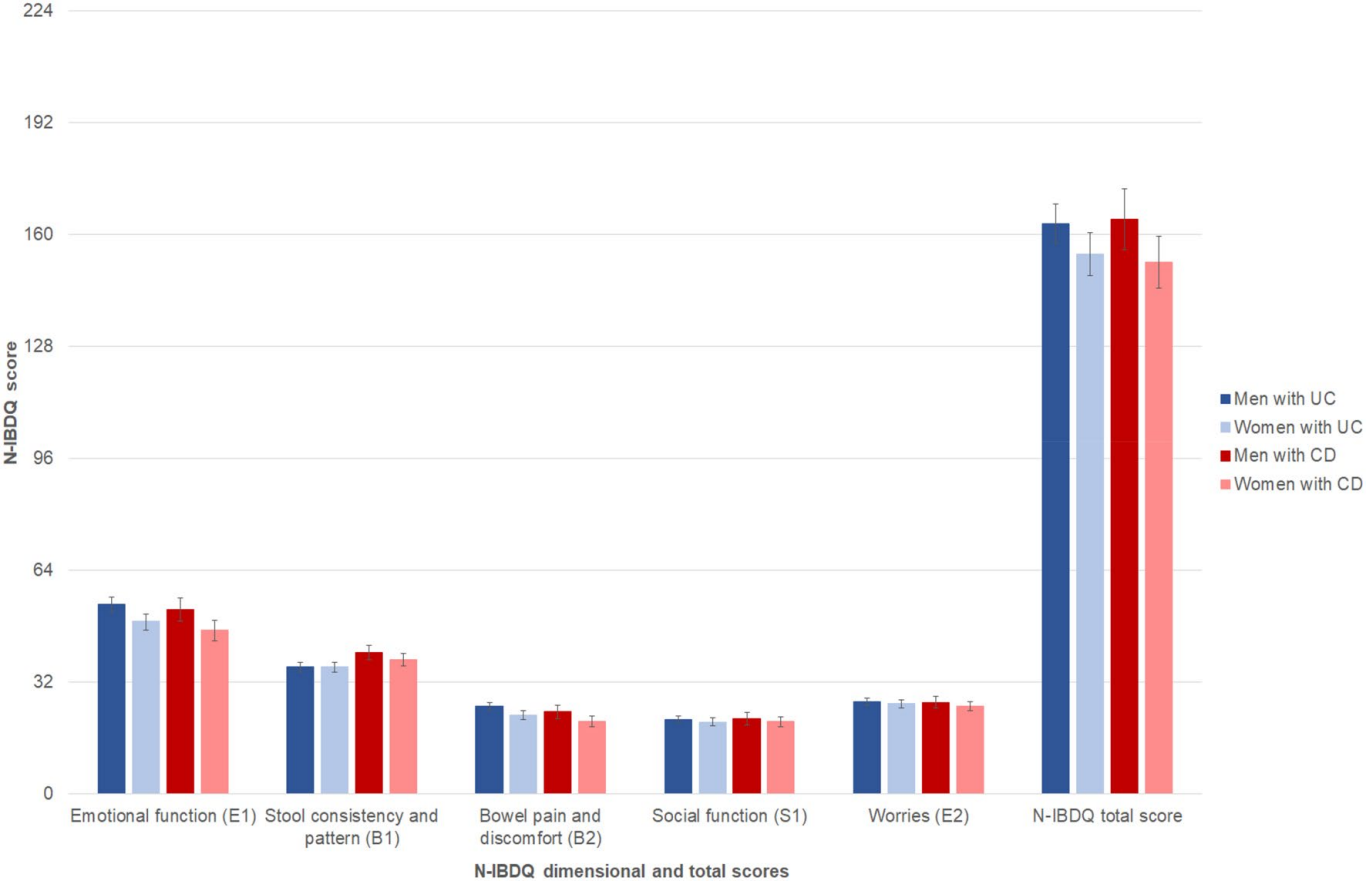
Mean Norwegian Inflammatory Bowel Disease Questionnaire dimensional and total scores in study patients, stratified by gender and diagnosis. Mean scores adjusted for age and educational status are shown, and error bars represent 95% confidence intervals

The dimensional and total N-IBDQ scores are presented in Table 2 and Fig. 4. Women with UC reported significantly lower scores in the E1 and B2 dimensions compared with men (Table 2). No statistical differences in the N-IBDQ total scores were observed between any of the groups.

### Factors associated with HRQoL

Table 3 lists the variables included in the multivariate regression models. Symptoms of depression, defined as HADS-D ≥ 8 and substantial fatigue, defined as FQ ≥ 4, were associated with statistically significant low SF-36 dimensional and N-IBDQ total scores for both patients with CD and UC (*p* < 0.01). Additionally, basic education only, living alone, HADS-A ≥ 8, and HBI ≥ 5 were associated with low SF-36 scores for patients with CD. For patients with UC, not employed/studying, HADS-A ≥ 8, and SCCAI ≥ 2.5 were significantly associated with low SF-36 scores (Table 3). The disease biomarkers CRP ≥ 5 mg/L and calprotectin > 250 µg/g were not associated with SF-36 or N-IBDQ scores for patients with CD, and only associated with significantly low N-IBDQ total scores for patients with UC. Higher GSE scores were associated with increased HRQoL in only one SF-36 dimension (GH) in patients with UC. Substantial fatigue had the greatest individual impact on N-IBDQ total scores in both patients with CD and UC, where N-IBDQ scores on average were 24 and 26 points lower for patients with CD (95% CI − 34 to − 12) and UC (95% CI − 33 to − 19), respectively, than for patients with FQ scores < 4 (Table 3). Symptom duration before diagnosis, which was measured in months, was not significantly associated with SF-36 or N-IBDQ scores in the univariate analysis and, therefore, not included in the multivariate regression.

**Table 3:** Estimated effect of explanatory variables on SF-36 dimensional scores and N-IBDQ total scores at the time of diagnosis in multiple linear regression, by diagnosis

	SF-36 dimension								
	PF	RP	BP	GH	VT	SF	RE	MH	N-IBDQ total score
*Crohn's disease (CD)*									
Substantial fatigue	− 13 [− 20 to − 5]	− 47 [− 64 to − 31]	− 27 [− 37 to − 16]	− 22 [− 31 to − 14]	− 33 [− 40 to − 25]	− 18 [− 29 to − 8]	− 24 [− 41 to − 7]	− 11 [− 17 to − 5]	− 24 [− 34 to − 12]
HBI for CD ≥ 5	− 13 [− 19 to − 6]	− 20 [− 35 to − 5]	− 16 [− 25 to − 6]	− 15 [− 22 to − 7]		− 15 [− 25 to − 6]			− 21 [− 31 to − 11]
HADS-depression ≥ 8						− 30 [− 43 to − 18]		− 19 [− 26 to − 12]	− 23 [− 36 to − 11]
HADS-anxiety ≥ 8								− 8 [− 14 to − 2]	
Living alone								− 8 [− 14 to − 2]	
Basic education only (≤ 12 years)					− 9 [− 16 to − 2]				
*Ulcerative colitis (UC)*									
Substantial fatigue	− 8 [− 13 to − 2]	− 36 [− 49 to − 24]	− 21 [− 29 to − 14]	− 18 [− 24 to − 11]	− 26 [− 32 to − 20]	− 13 [− 20 to − 5]	− 28 [− 41 to − 15]	− 8 [− 13 to − 4]	− 26 [− 33 to − 19]
HADS-depression ≥ 8	− 14 [− 22 to − 7]		− 20 [− 30 to − 9]			− 19 [− 29 to − 8]		− 19 [− 24 to − 13]	− 22 [− 31 to − 12]
Not employed/not studying	− 12 [− 21 to − 4]	− 27 [− 47 to − 8]				− 19 [− 31 to − 7]			
SCCAI for UC ≥ 2.5						− 12 [− 20 to − 5]			− 17 [− 24 to − 10]
HADS-anxiety ≥ 8								− 7 [− 12 to − 2]	− 15 [− 23 to − 7]
Age, by increasing year				0.3 [0.1–0.6]					
GSE, for every 1 unit increase				0.8 [0.2–1.3]					
Calprotectin > 250 µg/g									− 11 [− 18 to − 4]

Unstandardized regression coefficients (β) statistically significant to *p* < 0.01 are shown with 95% confidence intervals in brackets. Values of β represent estimated effects of the explanatory variables on SF-36 dimensional scores and N-IBDQ total scores

Variables insignificant in the regression model, and not shown: female gender (CD/UC), CRP (CD/UC), age (CD), not employed/studying (CD), GSE (CD), calprotectin (CD), basic education only (UC), Montreal disease severity ≥ 2 (UC)

*PF* physical function, *RP* role physical, *BP* bodily pain, *GH* general health, *VT* vitality, *SF* social functioning, *RE* role emotional, *MH* mental health, *HADS* Hospital Anxiety and Depression Scale, *GSE* General Self-Efficacy Scale, *HBI* Harvey-Bradshaw Index, *SCCAI* Simple Clinical Colitis Activity Index, *CRP* C-reactive protein

## Discussion

Our study is the first to compare HRQoL data in newly diagnosed patients with IBD from an unselected population-based inception cohort with HRQoL data from a reference population. Both men and women with CD and UC reported significantly lower scores in seven of the eight SF-36 dimensions than the representative sample of the Norwegian population. These findings were also clinically important, as shown by the moderate-to-large Cohen’s *d* effect sizes, except for BP and RE for men with UC, and PF for both sexes and diagnoses. Furthermore, we found few significant correlations between elevated levels of the disease biomarkers, calprotectin and CRP, and HRQoL in this study.

Most HRQoL data from patients with IBD originate from cross-sectional or cohort studies consisting of selected patients who have lived with IBD for months or years, are already on treatment, or are in remission [4, 5]. To the best of our knowledge, only two unselected population-based cohort studies have provided baseline HRQoL data for patients newly diagnosed with IBD. In an international multicenter study, Burisch et al. [5] found an improvement in HRQoL during the first year of disease in patients with CD and UC, although the factors associated with reduced HRQoL at the time of diagnosis were not evaluated. McCombie et al. [6] performed a smaller prospective observational study and found improvements in HRQoL six months after diagnosis. Unlike our study, neither study compared the HRQoL data in the study cohort to a reference population at the time of diagnosis. Additionally, both studies used the Short Form 12, consisting of 12 questions, and Short Inflammatory Bowel Disease Questionnaire, consisting of ten questions, to assess HRQoL, neither of which was directly comparable to the SF-36 and IBDQ questionnaires used in our study [5, 6].

Previous research has shown that HRQoL in patients with chronic disease, including IBD, tends to improve over time with treatment, especially in patients who achieve disease remission [1, 33–35]. Additionally, patients with chronic diseases will often change their perception of HRQoL over time, often referred to as a “response shift.” As outlined by Sprangers and Schwartz [36], a response shift can be defined as “a change in the meaning of one’s self-evaluation of [Quality of life] as a result of changes in internal standards, values and the conceptualization of [Quality of life].” As patients adapt to chronic diseases, a response shift can occur, which influences how they perceive and report their quality of life. Moreover, a person’s own mechanisms (coping ability, social support, and behavioral processes) and antecedents (personality, sociodemographic traits, and gender) influence the response shift process [36].

The patients in our study were newly diagnosed with a chronic disease, which likely contributed to lower HRQoL scores compared to those reported in previous studies, where HRQoL was measured later in the course of IBD disease [1, 33, 34]. Nevertheless, our study found moderate-to-large effect sizes in almost all SF-36 dimensions compared to the reference population, indicating that these changes are of clinical importance. Our data did not provide evidence that pre-diagnostic symptom duration was associated with SF-36 or N-IBDQ scores. A possible explanation could be that the study was designed such that patients were given access to the questionnaires on the day of inclusion, which often coincided with the date of diagnosis. Therefore, patients may not have had sufficient time to adapt to or accommodate their conditions. Nevertheless, our results could potentially overestimate the HRQoL in patients with long-standing symptom duration.

Substantial fatigue was significantly associated with lower HRQoL in all eight SF-36 dimensions and total N-IBDQ score. HADS depression score ≥ 8 was also associated with reduced HRQoL in several SF-36 dimensions and N-IBDQ total score reported by patients with CD and UC. Depression and fatigue were independently correlated with lower HRQoL scores [37, 38], although distinguishing measurements of fatigue from depression remains difficult because symptoms of fatigue may also lead to depressive symptoms [37]. Our findings coincide with results from a recent study showing that tiredness/exhaustion/fatigue and anxiety/low mood/depression are major symptoms in patients with both CD and UC [39]. An ongoing longitudinal study of the current study population will hopefully determine whether these correlations remain consistent over time.

Surprisingly, we found few significant correlations between the disease biomarkers and low HRQoL. D’Haens et al. [14] found that calprotectin levels were the best surrogate marker for mucosal inflammation and a cut-off value of 250 µg/g can indicate “significant mucosal inflammation.” In our study, we found median calprotectin values of 242 and 369 µg/g for patients with UC and CD, respectively; therefore, we expected significant associations between this disease marker and reduced HRQoL. However, in the multivariate regression analysis, elevated levels of calprotectin > 250 µg/g and CRP levels ≥ 5 were not associated with worsened HRQoL in patients with CD. In patients with UC, calprotectin levels > 250 µg/g were associated with low HRQoL scores as measured by the N-IBDQ, but not in any of the SF-36 dimensions. Therefore, our results suggest that symptom burden, as measured by disease activity indices and psychosocial measures, may have a more pronounced impact on HRQoL than clinical disease biomarkers.

This study had some limitations. First, the observational design does not allow the testing of causal relationships or generation of causal conclusions. Second, only 370 of the 573 patients (64.5%) completed the PROM questionnaire, reducing the size of our study population. However, few statistically significant differences were observed between those who answered the PROMs and those who did not. Therefore, we conclude that the study population was representative. Third, the number of men and women with CD was small compared with the reference population. Although the mean HRQoL scores and corresponding SD for the reference population were available, the original data for the reference population were not. Given the unequal group sizes, we were unable to test the assumption of homogeneity of variance. Instead, we compared the SDs from each patient group to their corresponding reference populations. This revealed differences in the two SF-36 dimensions RP and RE, enabling potential type I errors in analysis of the RP and RE dimensions. Thus, gender-stratified results from these two dimensions should be interpreted with caution. Finally, our study lacked a validated, objective measure of endoscopic disease severity in patients with CD. Instead, a shorter version of the SES-CD was used, which has not been validated in other studies, diminishing our ability to assess the association between endoscopic CD severity and HRQoL. Nevertheless, we included a high calprotectin level > 250 µg/g as an explanatory variable. This cutoff value for calprotectin has previously been shown to correlate significantly with endoscopic disease activity in CD and recommended as a surrogate marker [14].

## Conclusion

Newly diagnosed patients with CD and UC reported statistically and clinically significantly lower scores in seven of the eight SF-36 dimensions compared to a representative sample of the Norwegian population. In patients with CD and UC, high HADS depression scores, substantial fatigue, and high clinical activity scores (SCCAI and HBI scores, respectively) were associated with reduced HRQoL. Additionally, not being employed or studying was associated with reduced HRQoL in patients with UC. Surprisingly, we found few significant correlations between reduced HRQoL and disease biomarkers, as indicated by elevated calprotectin and CRP levels. This study provides real-world HRQoL data that reflects the burden of IBD disease in newly diagnosed patients.

## Data Availability

The data supporting this study are stored on a secure server for sensitive data managed by the University of Oslo, and cannot be shared publicly due to legal and privacy concerns. Access to anonymized data is subject to approval after a reasonable request is made to the corresponding author.
